# Differential Pathogenicity of SHIV KB9 and 89.6 Env Correlates with Bystander Apoptosis Induction in CD4+ T cells

**DOI:** 10.3390/v11100911

**Published:** 2019-10-01

**Authors:** Tugba Mehmetoglu-Gurbuz, Anjali Joshi, Himanshu Garg

**Affiliations:** Center of Emphasis in Infectious Diseases, Department of Molecular and Translational Medicine, Texas Tech University Health Sciences Center, El Paso, TX 79905, USA; tugba.gurbuz@ttuhsc.edu (T.M.-G.); anjali.joshi@ttuhsc.edu (A.J.)

**Keywords:** HIV, SHIV, KB9, 89.6, bystander apoptosis, CXCR4, CCR5, apoptosis inducing potential, Envelope, pathogenesis, Caspase

## Abstract

SHIV variants KB9 and 89.6 show differential pathogenesis in primate models with KB9 causing rapid CD4 decline while 89.6 failing to induce disease. We attempted to determine whether the differential pathogenicity of KB9 versus 89.6 was a result of differential bystander apoptosis inducing potential (AIP) of the Env glycoproteins from these viruses. We find that the KB9 Env was highly potent at inducing bystander apoptosis in CD4+ target cells compared to 89.6 Env. Cell death induction by KB9 showed classical signs of apoptosis including mitochondrial depolarization, caspase activation and PARP cleavage. Inhibiting Env mediated fusion by T20 peptide inhibited KB9 mediated bystander apoptosis. KB9 and 89.6 differed in terms of co-receptor usage with 89.6 preferring CXCR4 while KB9 using both CXCR4 and CCR5 with equal efficiency. Our study suggests that higher bystander AIP of KB9 Env compared to 89.6 may be the basis for the differential pathogenesis of these viruses.

## 1. Introduction

HIV-1 infection in humans leads to a selective depletion of CD4+ T cells leading to immunodeficiency. While the outcome of HIV-1 infection in humans is immunodeficiency in most cases; the outcome of natural infection in primates is strikingly different [1]. Natural infection in Sooty Mangabeys and African Green Monkeys with SIV_sm_ and SIV_agm_ variants respectively fails to induce CD4 loss or AIDS development [2]. However, Rhesus Macaques infected with SIV_mac_ virus are known to succumb to AIDS like illness albeit at different frequency than humans [3,4]. The lack of a suitable animal model that recapitulates the immunopathogenic outcome of HIV resulted in the development of the chimeric SHIV model [5,6].

The chimeric SHIV model has been used extensively for pathogenesis studies and the SHIV 89.6 and KB9 are well established model systems to study differential pathogenesis of viral variants [7]. While the original SHIV 89.6 virus replicated to high levels in Rhesus Macaques, it failed to induce AIDS like illness. Passage of SHIV89.6 in Rhesus Macaques resulted in generation of the pathogenic variant designated as SHIV-89.6P [6]. SHIV-KB9 is the molecular clone of this pathogenic virus isolated from Rhesus Macaques which has been shown to be highly pathogenic and results in CD4 loss within several months [8]. The differential pathogenesis of these variants has been mapped to the Env glycoprotein with the membrane fusogenic activity directly associated with this phenomenon [9,10,11].

HIV-1 Env glycoprotein is a major determinant of HIV pathogenesis and bystander apoptosis mediated by HIV Env has been implicated in disease induced CD4 loss [12,13,14]. Variations in HIV Env glycoprotein phenotype in terms of bystander apoptosis inducing potential (AIP) have been associated with CD4 loss and disease progression [15,16]. Coincidentally, HIV Env mediated bystander apoptosis also correlates with Env fusogenic activity with a more specific role of hemifusion between membranes being implicated in this phenomenon [17,18]. Whether SHIV Env variants with altered pathogenicity in vivo also differ in AIP in vitro remains undetermined.

In this study, we determined whether differential pathogenicity of KB9 versus 89.6 was due to different bystander AIP of the Env glycoproteins from these viruses using an in vitro co-culture model. We find that KB9 Env causes significantly higher apoptosis in CD4 T cell lines as well as human and Rhesus peripheral blood mononuclear cells (PBMCs) that is dependent on membrane fusion and caspase activation.

## 2. Materials and Methods

### 2.1. Cells and Reagents

SupT1 cells (kindly provided by the NIH AIDS Reagent Program) were maintained in RPMI media supplemented with 10% FBS and penicillin streptomycin (5000 U/mL). HeLa and TZM-bl cells (NIH AIDS reagent program) were maintained in Dulbecco’s modified eagle’s medium (DMEM) supplemented with 10% FBS and penicillin streptomycin (5000 U/mL). The CCR5 expressing SupT cell line (SupT-R5-H6) has been described previously [19] and was maintained in RPMI with 10% FBS and 3 µg/mL blasticidin. Peripheral blood mononuclear cells (PBMCs) from Indian Rhesus Macaques were obtained from Fisher Scientific and cultured in RPMI supplemented with 20% FBS. Fusion inhibitor T20 (Enfuvirtide), CXCR4 antagonist AMD3100 and CCR5 antagonist Maraviroc were kindly provided by the NIH AIDS reagent program. Pan Caspase inhibitor Z-VAD-fmk and mitochondrial potential sensor dye DiOC_6_ were obtained from EMD Millipore.

### 2.2. Plasmid Constructs

Plasmids expressing YU-2, 89.6 and Lai Env have been described before [19]. pSHIV-KB9 3′ and SHIV 89.6P 3′ [8] were obtained from the NIH AIDS Reagent Program and used as template for cloning SHIV KB9 and SHIV 89.6 Env regions. Briefly, the *env* and *rev* regions of SHIV KB9 and SHIV 89.6 were PCR amplified with subtype B specific primers HZBIE: 5′ AGC TGG ATC CGT CTC GAG ATA CTG CTC CCA CCC 3′ and HZBIB: 5′ CAC CGA TCA AGC TTT AGG CAT CTC CTA TGG CAG GAA GAA G using the Phusion High Fidelity PCR kit (New England Biolabs, Ipswich, MA, USA). The amplified Env region was cloned into the pCDNA3.1 vector using the pCDNA3.1 directional TOPO^®^ Expression kit (Invitrogen, Carlsbad, CA, USA). Authenticity of the inserts was confirmed by sequencing.

### 2.3. Expression and Shedding of SHIV Envs

HeLa cells plated in 24 well plates at 10^5^ cells/well were transfected with different Env constructs using the TurboFect transfection Reagent (Thermofisher Scientific, Waltham, MA, USA). Twenty-four hours post-transfection, the media was removed and SupT1 or SuPT-R5-H6 cells were added to the cultures at 0.5 × 10^6^ cells per well. The cells were co-cultured for 24 h following which apoptosis was determined via different methods. The suspension cells were collected, stained with Annexin V (BD bioscience) and analyzed by flow cytometry using a Beckman Coulter Gallios Flow cytometer. For some assays, apoptosis was detected by staining with mitochondrial membrane potential sensitive dye DiOC_6_ (10nM) followed by flow cytometry. At least 10,000 events were acquired and analyzed using the Flow Jo software (Tree Star). HIV inhibitors T20, AMD3100 and Maraviroc were added at the time of co-culture, while z-VAD-fmk was incubated with SupT cells for 30 min prior to addition to HeLa cells expressing the different Envs. For measurement of cell surface Env expression, HeLa cells transfected in a 96-well plate were stained with anti-Env antibody (b12, kindly provided by the NIH AIDS reagent program) in RPMI-10 medium followed by staining with secondary antibody anti-human alexa-594. Nuclei were visualized using DAPI staining and images acquired using the NikonTi fluorescent microscope. For quantitation of Env expression, entire wells (*N* = 4) of the 96-well plate were scanned using the Cytation5 imager (Biotek, Winooski, VT, USA) and mean fluorescent intensity (MFI) of staining, cell count and object sum area was calculated using the Gen5 software.

For Env shedding, transfected HeLa cells were cultured overnight in RPMI medium lacking Met and Cys and supplemented with 10% FBS and [35S] Met/Cys. Cell lysates and culture supernatants were immunoprecipitated with HIV-Ig (kindly provided by the NIH AIDS reagent program) coated protein A beads. Immunoprecipitated complexes were washed, resolved by SDS-PAGE followed by PhosphorImager analysis.

### 2.4. Measurement of Apoptosis Induction

For apoptosis induction in primary cells, cryopreserved PBMCs from healthy donors were used. Unstimulated PBMCs were cocultured with HeLa cells transfected with either SHIV KB9 or 89.6 Env for 48h. The suspension cells were collected and stained with an apoptosis panel comprising of the following antibodies: CD3-Cy7, CD4-Tx Red, CD8-APC (Beckman Coulter) along with CaspACE FITC-VAD-FMK (Promega, Madison, WI, USA) as described previously [16]. Stained cells were washed and fixed using IOTest 3 Fixative Solution (Beckman Coulter) and assayed by flow cytometry. At least 20,000 events for each sample were acquired. Data was analyzed using FlowJo software (Tree Star). Cells were first gated on the CD3+ population and apoptosis in CD4+ and CD8+ T cell subsets was determined along with the CD4:CD8 ratio. Apoptosis induction in Rhesus PBMCs was measured as above and human antibodies with cross reactivity to Rhesus CD4 (BD Biosciences 562402) and CD3 antigens (BD Biosciences 557749) were used.

### 2.5. Pseudotyped Virus Studies

The 293T cells were transfected with the pNLLuc-R^−^/E^−^ [20] HIV backbone along with different Env constructs. Virus supernatants were harvested 48 h post-transfection, cleared of cellular components by centrifugation, aliquoted and stored at –80 °C. Pseudotyped virus stocks were used to infect the indicator TZM-bl cell line in the presence of 20µg/mL DEAE dextran (Sigma). Luciferase activity was determined 48 h or 72 h post infection using the BriteLite plus Luciferase assay substrate (PerkinElmer, Waltham, MA, USA) using FLUOstar Omega multi-mode microplate reader (BMG Labtech, Ortenberg, Germany). Each pseudotyped virus stock was titrated in TZM-bl cells and virus concentration in the linear range was used for experiments. Different inhibitors like T20, AMD3100 and Maraviroc were added at different concentrations at the time of infection.

### 2.6. Immunoblotting

Detection of full length and cleaved PARP was achieved via Western blotting. SupT1 cells collected from the coculture experiments were lysed with RIPA buffer (1×) supplemented with protease inhibitors. Samples were boiled at 92 °C for 6 min, resolved on a 4–12% SDS-PAGE gel followed by transfer onto PVDF membranes (Millipore). Following transfer, the membrane was blocked with 5% skim milk solution for 1 h at room temperature. Blots were incubated with primary anti-PARP antibody (Cell signaling #9542) overnight at 4 °C followed by secondary anti-rabbit HRP antibody for 1h at room temperature. Bands were visualized using the SuperSignal™ West Pico enhanced chemiluminescence detection reagent (Thermofisher Scientific, Waltham, MA, USA) and quantitated using the GeneTools software (SYNGENE, New Castle, England).

### 2.7. Statistical Analysis

Statistical analysis was conducted using the GraphPad Prism software (San Diego, CA). Annexin-V and DiOC_6_ staining data, cell surface staining using B12 antibody and co-receptor usage determination were analyzed utilizing one-way ANOVA followed by Dunnett’s multiple comparison test. Data from T20 and ZVAD-FMK experiments was analyzed using two tailed unpaired student’s t-test. Human PBMC co-culture data was analyzed using the Wilcoxon matched-pairs signed rank test and Rhesus Macaque co-culture data was analyzed with one-way ANOVA followed by Dunnett’s multiple comparison test.

## 3. Results

### 3.1. HIV KB9 is More Potent at InducingBbystander Apoptosis in SupT1 Cell Lines in Coculture Model

**Figure 1:**
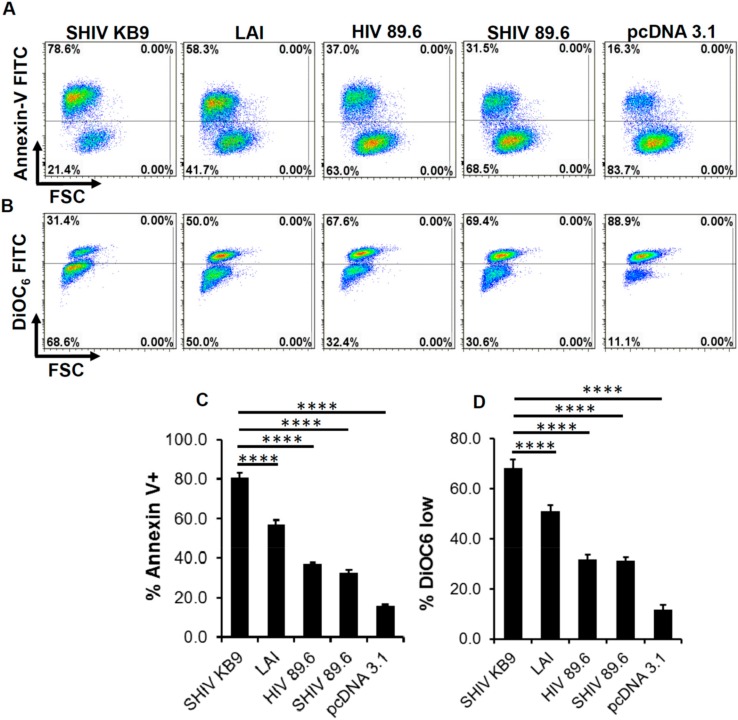
SHIV KB9 Env mediates higher apoptosis in bystander CD4+ T cells. HeLa cells were transfected with pSHIV-Env (KB-9 or 89.6), pHIV-1-Env (89.6 or Lai) or control vector pcDNA3.1 and co-cultured with the SupT1 cell line. Apoptosis was detected either via annexin V staining or DiOC6 staining 24 hours post co-culture. Representative dot plots for annexin V (**A**) and DiOC6 staining (**B**) are shown. Apoptosis induction by different Envs as determined by Annexin V (**C**) or DiOC6 staining (**D**) is shown. Data are mean ± SD of triplicate observations. *p* ≤ 0.0001 as determined by one-way ANOVA followed by Dunnett’s multiple comparison test. All experiments were repeated with similar results.

Previously, we and others have studied HIV Env mediated bystander apoptosis in coculture models whereby HIV Env expressing cells (effectors) induce apoptosis in CD4+ T cells (target) [18,21,22,23,24]. Studies from these experiments have shown that the differential in vitro AIP of both HIV Env mutants and primary Env variants correlates with CD4 loss in the humanized mice model as well as in HIV patients [15,16,17,25]. To determine whether KB9 and 89.6 Envs have differential AIP we utilized the same coculture model system. SHIV KB9 and 89.6 *env* and *rev* regions were cloned into pcDNA3.1 expression vector and used to transfect HeLa cells which were then cocultured with SupT1 cells. AIP of the respective Envs was determined 24 h post coculture by measuring apoptosis markers like mitochondrial membrane depolarization or phosphatidyl serine (PS) exposure on the cell surface. Demonstrated in Figure 1 is the flow cytometry analysis of bystander apoptosis mediated by KB9, Lai, HIV-89.6 and SHIV-89.6 Envs in SupT cells determined either via Annexin V staining (Figure 1A) or mitochondrial depolarization measured via DiOC_6_ staining (Figure 1B). We found that KB9 shows higher AIP compared to SHIV 89.6 as well as Lai Env, used as positive control, both in Annexin V (Figure 1C) and DiOC6 staining (Figure 1D). As HIV 89.6 and SHIV 89.6 have minor sequence variation in the 5′ and 3′ regions of the genome, we also compared the two Envs in their capacity to induce bystander apoptosis in our assay and found similar results. These data suggest that SHIV KB9 Env, at least in vitro, is fundamentally more pathogenic as a result of higher AIP compared to SHIV 89.6.

### 3.2. Bystander Apoptosis Induction Mediated by SHIV-KB9 Env is gp41 Dependent

**Figure 2:**
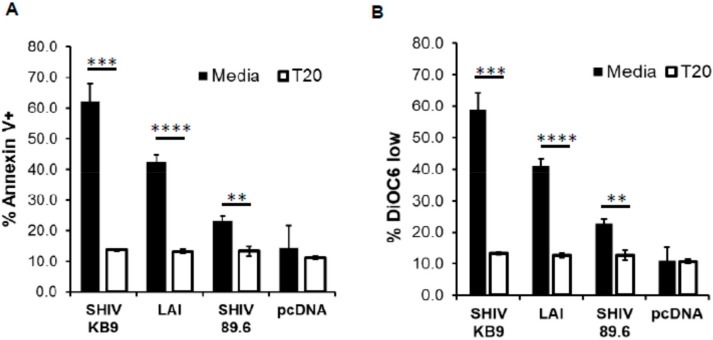
HIV gp41 inhibitor T20 inhibits SHIV KB9 mediated bystander apoptosis. HeLa cells transfected with different Env constructs or control vector were cocultured with SupT cells in the presence or absence of T20 (2 μM). Apoptosis was detected 24 h post co-culture using Annexin-V staining (**A**). Mitochondrial depolarization was detected via DiOC6 staining followed by flow cytometry analysis (**B**). Data are mean ± SD of triplicate observations. **** = *p* ≤ 0.0001; *** = *p* ≤ 0.001; ** = *p* ≤ 0.01 as determined by two tailed unpaired t-test. All experiments were repeated with similar results.

HIV Env is composed of two subunits. The gp120 surface unit binds to the CD4 receptor and the coreceptor, while the transmembrane gp41 subunit mediates fusion of viral and cellular membranes. This leads to cell to cell fusion or hemifusion in coculture experiments when Env expressing cells come in contact with CD4/co-receptor expressing cells [26]. Hemifusion mediated by the gp41 subunit is required for HIV Env mediated bystander apoptosis which has been demonstrated by the use of gp41 inhibitors like C34 and T20 [18,22] that also prevent apoptosis. Interestingly, the membrane fusogenic activity of KB9 is significantly higher than SHIV 89.6 and has been implicated in their variable pathogenesis [9,27]. We hence tested whether the bystander apoptosis induction by SHIV KB9 in our model was gp41 dependent by using the T20 fusion inhibitor. As seen in Figure 2, SHIV KB9 mediated bystander apoptosis was completely inhibited by gp41 fusion inhibitor T20 as measured by Annexin V (Figure 2A) or DiOC_6_ staining (Figure 2B). This inhibition was similar to that seen with Lai and SHIV 89.6 Envs in the presence of T20 while expression of control vector pCDNA3.1 did not alter background apoptosis in the presence of T20 suggesting that the phenomenon was specific to HIV Env. These data demonstrate that AIP of SHIV KB9 Env is dependent on gp41 function and is fundamental to bystander apoptosis induction. This is also consistent with findings by Labonte et al. whereby membrane fusion activity of KB9 was associated with cytopathic effect [27].

### 3.3. Differences in Bystander Apoptosis Between the KB9 and 89.6 Envs are Not Due to Differences in Env Expression or Env Shedding

**Figure 3:**
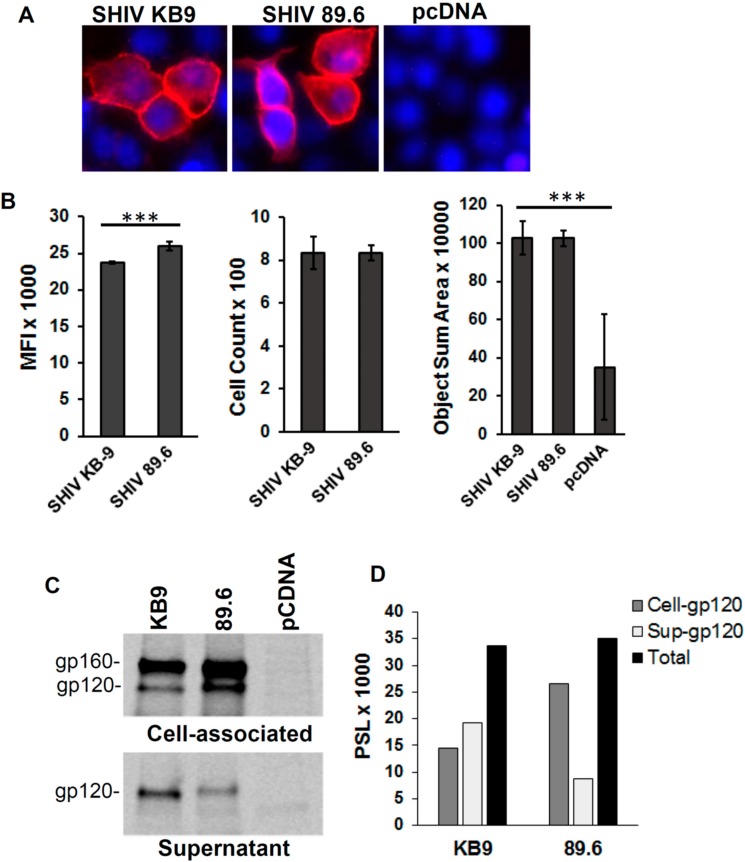
Env expression and Env shedding for KB9 and 89.6 Envs. HeLa cells were transfected with Env expression constructs for KB9, 89.6 or control pcDNA3.1 vector. Surface Env expression was determined 48 hrs post transfection after staining with the b12 antibody flowed by fluorescence microscopy (**A**). Whole wells of 96-well plate were scanned to determine MFI, positive cell counts and object sum area. *p* ≤ 0.0001 as determined by one-way ANOVA (**B**). HeLa cells were transfected as above and Env shedding was determined after radiolabeling of cells with [35S] Met/Cys followed by immunoprecipitation of cell lysates and supernatants using HIV Ig (**C**). Quantitation of cell associated and shed gp120 (**D**). One representative of two independent experiments is shown.

It is conceivable that differences in Env expression between the KB9 and 89.6 Envs were responsible for the higher apoptosis seen in SHIV KB9 Env expressing co-cultures. We hence determined whether differences in AIP by KB9 and 89.6 Envs were due to different transfection efficiencies of the two constructs or different levels of Env expression after transfection with the KB9 and 89.6 Env expression constructs. For this, HeLa cells were transfected using the same protocol as for apoptosis assays and surface Env expression was determined after staining with the Env binding b12 antibody. As demonstrated in Figure 3A, cell surface Env expression was comparable after transfection of HeLa cells with SHIV KB9 and the SHIV 89.6 Env constructs. Analysis of cells from whole wells (*N* = 4) of a 96-well plate also revealed no differences between the number of Env expressing cells and the sum of the Env positively staining area (Figure 3B). However, there was a significant difference between the MFI of Env staining between SHIV KB9 and SHIV 89.6 transfected wells (Figure 3B).

Further, we assessed the role of Env shedding in KB9 and 89.6 expressing cultures and if that translated to differences in apoptosis induction by the two Envs. As demonstrated in Figure 3C, minor differences were visible between cell associated and shed Env between the two Envs. The KB9 Env was slightly less associated with the cells with more shedding into the supernatants. However, the total functional Env (gp120) expression in the cell lysates and culture supernatants was similar for both KB9 and 89.6 Envs (Figure 3D). This Env shedding data correlates well with fluorescent microscopy data in Figure 3B where SHIV 89.6 shows higher MFI than SHIV KB9. Overall these data suggest that the differences in bystander apoptosis seen between KB9 and 89.6 Envs are not due to differences in transfection, Env expression levels or Env shedding.

### 3.4. SHIV 89.6 Prefers CXCR4 Co-Receptor Usage While KB9 Utilizes CXCR4 or CCR5 with Equal Efficacy for Viral Entry

**Figure 4:**
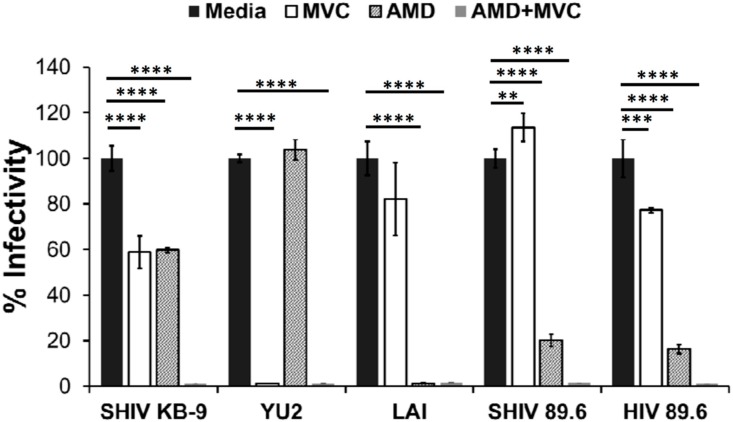
Variable coreceptor usage by SHIV Envs. HIV-1 virions pseudotyped with indicated Envs were used to infect TZM-bl indicator cell line. Pseudotyped virus infections were conducted in the presence of inhibitors Maraviroc (MVC), AMD 3100 (AMD), a combination of both AMD and MVC (AMD + MVC) or no inhibitor (media). Virus infectivity was determined 72 h later by measuring luciferase activity. **** = *p* ≤ 0.0001; *** = *p* ≤ 0.001; ** = *p* ≤ 0.01 as determined by one-way ANOVA followed by Dunnett’s multiple comparison test.

Both SHIV-89.6 and KB9 are dual tropic viruses known to be capable of utilizing either CXCR4 or CCR5 for viral entry [28]. However, it is unclear whether these viruses prefer one of the two receptors when both receptors are available on cells. We hence conducted pseudotyped virus infection studies in the presence of AMD3100 (CXCR4 antagonist), Maraviroc (CCR5 antagonist) or a combination of both to determine the co-receptor preference of the two Envs. As demonstrated in Figure 4, SHIV 89.6 was not inhibited by Maraviroc (MVC) but almost completely inhibited by AMD3100. The results were similar for HIV 89.6 suggesting these two Envs are phenotypically similar even with the differences in the 5′ and 3′ regions. Interestingly, a combination of both AMD3100 (AMD) and MVC inhibited SHIV 89.6 and HIV 89.6 pseudotyped viruses completely. On the other hand, neither AMD nor MVC were able to inhibit KB9 more than 50% but a combination of both the antagonists inhibited the virus completely. Lai and YU-2 yielded results consistent with their dependence on CXCR4 or CCR5 receptors respectively. These data suggest that while SHIV-89.6 Env prefers to use CXCR4 for entry, KB9 utilizes either CXCR4 or the CCR5 co-receptor with similar efficiency. These differences in CCR5 binding affinity for KB9 versus 89.6 have been reported previously [9] and is consistent with our data.

### 3.5. SHIV-KB9 Env Utilizes both CCR5 and CXCR4 Equally for Apoptosis Induction

**Figure 5:**
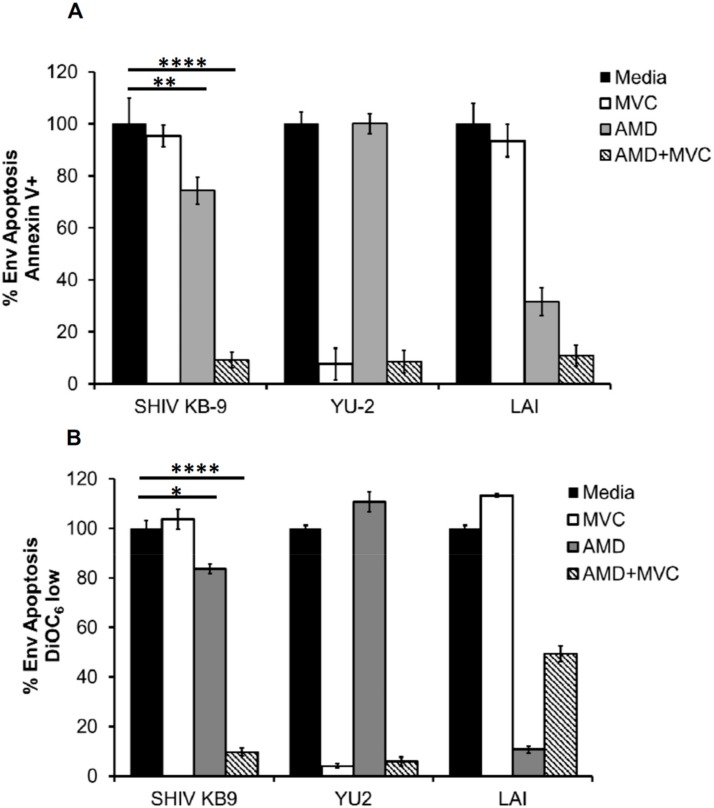
SHIV KB9 uses both CXCR4 and CCR5 equally for induction of bystander apoptosis. HeLa cells transfected with various Envs were cocultured with SupT-R5-H6 cells. Coreceptor inhibitors AMD3100 (CXCR4) and MVC (CCR5) were used to inhibit apoptosis either alone or in combination (AMD + MVC). Apoptosis was determined 24 h later via annexin V staining (**A**) or detecting mitochondrial depolarization via DiOC6 staining (**B**). Data was normalized for each Env and percent Env specific apoptosis was calculated. Data are mean ± SD of triplicate observations. **** = *p* ≤ 0.0001; ** = *p* ≤ 0.01 and * = *p* ≤ 0.05 as determined by one-way ANOVA followed by Dunnett’s multiple comparison test. All experiments were repeated with similar results.

Our data above demonstrated that the KB9 Env utilizes CXCR4 or CCR5 with equal efficacy for viral entry. As KB9 is a potent inducer of bystander apoptosis, we asked whether KB9 selectively utilizes CXCR4 or CCR5 for induction of apoptosis in our above described coculture model system. For the HeLa-Env and T cell coculture experiment we utilized the SupT-R5-H6 cells as targets that are engineered to express CCR5 along with constitutive expression of CXCR4 [19]. As seen in Figure 5, AMD3100 or MVC when used alone, failed to inhibit KB9 mediated bystander apoptosis completely as evident via Annexin V (Figure 5A) or DiOC_6_ (Figure 5B) staining. However, a combination of both AMD and MVC inhibited apoptosis completely (Figure 5A,B). This suggests that KB9 can utilize either CXCR4 or CCR5 for induction of bystander apoptosis similar to the results seen with virus entry above.

### 3.6. SHIV KB9 Mediated Bystander Apoptosis is Partially Caspase Dependent

**Figure 6:**
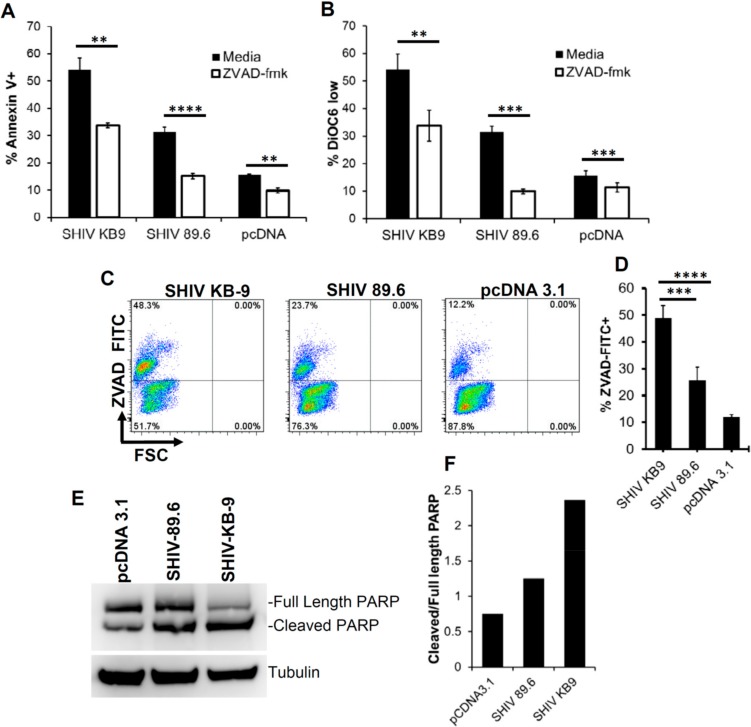
Bystander apoptosis mediated by SHIV KB9 is caspase dependent. HeLa cells expressing SHIV Env were cocultured with SupT cell in the presence or absence of pan caspase inhibitor ZVAD-fmk. Apoptosis was detected 24 h later using Annexin V (**A**) or DiOC6 staining (**B**). **** = *p* ≤ 0.0001; *** = *p* ≤ 0.001; ** = *p* ≤ 0.01 as determined by two tailed unpaired t-test. Activation of caspases in HeLa Env-SupT cocultures was detected by staining with ZVAD-FITC. Representative dot plots are shown (**C**) and data from triplicate observations is shown in (**D**). **** = *p* ≤ 0.0001; ** = *p* ≤ 0.01 as determined by one-way ANOVA followed by Dunnett’s multiple comparison test. PARP cleavage was detected in SupT cells cocultured with HeLa cells transfected with different SHIV Envs or control vector. Western blot of PARP protein levels and tublin control is shown in (**E**). Quantitation of PARP cleavage as ratio of cleaved PARP/total PARP is shown in (**F**).

The signaling pathway involved in HIV Env and cellular interactions resulting in apoptosis remains debated. The role of classical apoptosis pathways including caspase 3 activation has been demonstrated in this process by us and others [22,23]. We hence asked whether KB9 Env mediated bystander apoptosis involves activation of the classical pathways of apoptosis. For this, we tested whether KB9 and 89.6 Env mediated apoptosis was inhibited by Z-VAD-fmk, a pan caspase inhibitor in our co-culture model system. Apoptosis mediated by SHIV Envs was reduced but not completely inhibited by the pan caspase inhibitor Z-VAD-fmk both in Annexin V staining (Figure 6A) and DiOC_6_ staining (Figure 6B). We next tested whether this apoptosis involved activation of caspases by utilizing ZVAD-FITC staining that binds to active caspases and detects cells undergoing apoptosis. Here again we found that KB9 Env induced higher number of active caspase + bystander cells than 89.6 (Figure 6C and D). One of the most prominent downstream markers of classical apoptosis is PARP cleavage by caspases. We hence tested whether KB9 mediated apoptosis involved PARP cleavage and compared this phenomenon to 89.6 Env. As seen in Figure 6E, KB9 Env showed higher levels of cleaved PARP compared to 89.6. A quantitation of cleaved PARP to total PARP (Figure 6F) further validated the increased AIP of KB9 Env. These studies support the hypothesis that KB9 Env induces increased bystander apoptosis in CD4 target cells via a classical apoptosis pathway involving caspase activation, PARP cleavage, mitochondrial membrane depolarization and phosphatidyl serine (PS) exposure.

### 3.7. Coculture of SHIV KB9 Env Expressing Cells with Unstimulated PBMCs Results in a Specific Loss of CD4+ T cells Via Apoptosis

**Figure 7:**
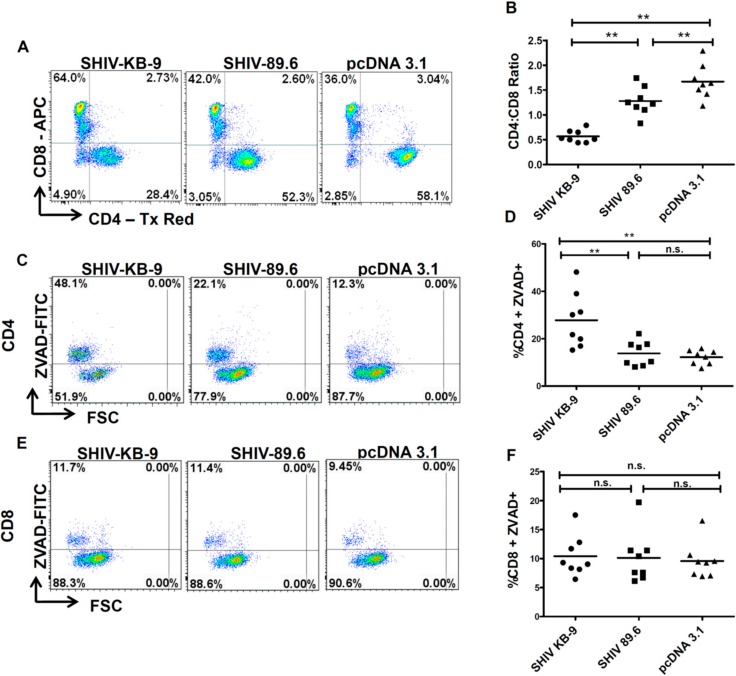
SHIV KB9 Env caused specific loss of CD4 cells via apoptosis in PBMC from healthy donors. HeLa cells were transfected with SHIV Envs (KB-9 and 89.6), or control vectors (pcDNA3.1) and co-cultured with PBMCs from healthy donors. At 48-hours post co-culture, PBMCs were collected and stained for CD3, CD4, CD8 and ZVAD-FITC and analyzed by flow cytometry. Cells gated on CD3+ population were analyzed for the CD4:CD8 ratio. Representative dot plots of the CD4:CD8 ratio are shown in (**A**) and results from eight different donors are shown in (**B**). Apoptosis was detected as ZVAD-FITC+ cells in CD3 + CD4 + population. Representative dot plots of CD4 apoptosis are shown in (**C**) and results from 8 different donors are shown in (**D**). Apoptosis was detected as ZVAD – FITC + cells in CD3 + CD8 + population. Representative dot plots of CD8 apoptosis are shown in (**E**) and results from eight different donors are shown in (**F**). Wilcoxon matched-pairs signed rank test was used for statistical analysis (** = *p* ≤ 0.01).

To further assess the relevance of differential AIP between SHIV 89.6 and KB9 Envs to differential pathogenesis, we conducted co-culture experiments using human PBMCs from healthy donors as target cells. As total PBMCs comprise of both CD4 and CD8 T cells, this model also provides the advantage of analyzing the specificity of apoptosis induction in CD4 cells and determining changes in the CD4:CD8 ratio [16]. As demonstrated in Figure 7, co-culture of unstimulated PBMCs with KB9 Env expressing cells resulted in a dramatic loss of CD4 cells with a significant reduction in the CD4:CD8 ratio (Figure 7A,B) compared to 89.6 Env or the pcDNA 3.1 control. Further analysis of apoptosis in different cell populations was conducted using ZVAD-FITC staining that binds to active caspases. Cells were gated for CD3 + CD4+ or CD3 + CD8 + apoptosis in each population determined by ZVAD-FITC staining. As seen in Figure 7C, we saw a marked increase in CD4 apoptosis in KB9 cocultures compared to 89.6 Env or the pcDNA3.1 control. Interestingly, there was minimal apoptosis detected in CD8 cells (Figure 7D,E) in all of the cocultures, further validating the specificity of Env mediated apoptosis for CD4 cells. These results also suggest that the inversion of the CD4:CD8 ratio seen in Figure 6A was a consequence of specific loss of CD4 cells via apoptosis. A total of eight different PBMC donor samples were tested for reproducibility which showed significant differences in the CD4:CD8 ratio (Figure 7B) and CD4 apoptosis (Figure 7D) but there was no difference in CD8 apoptosis between KB9 and 89.6 Envs (Figure 7F). These findings indicate that specific apoptosis of CD4 cells mediated by KB9 Env may be responsible for the higher pathogenesis of this viral variant.

### 3.8. Coculture of SHIV KB9 Env Expressing Cells with Rhesus PBMCs Also Results in a Specific Loss of CD4 + T cells Via Apoptosis

**Figure 8:**
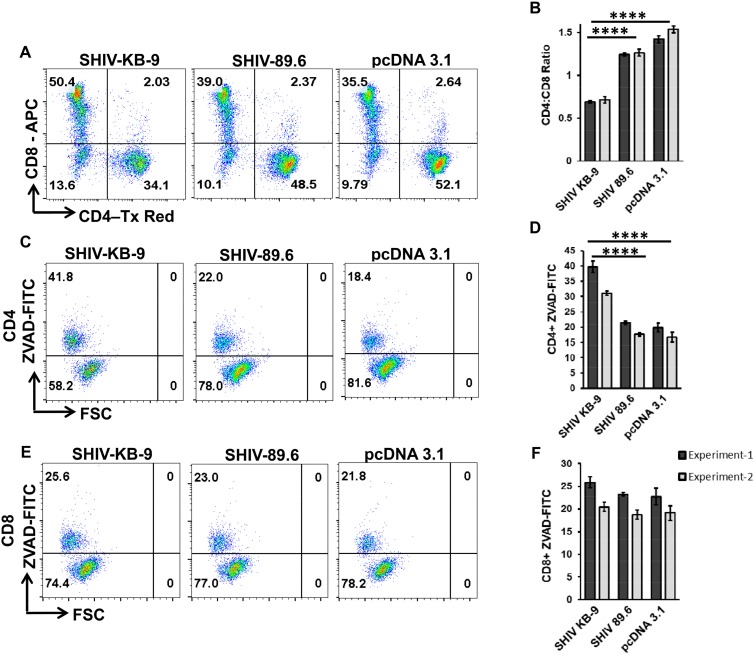
SHIV KB9 Env causes specific loss of CD4 cells via apoptosis in PBMC from Rhesus Macaques. HeLa cells were transfected with SHIV Envs (KB-9 and 89.6), or control vector (pcDNA3.1) and co-cultured with PBMCs from Indian Rhesus Macaques. At 48-hours post co-culture, PBMCs were collected and stained for CD3, CD4, CD8 and ZVAD-FITC and analyzed by flow cytometry. Cells gated on CD3+ population were analyzed for the CD4:CD8 ratio. Representative dot plots of the CD4:CD8 ratio are shown in (**A**) and average ± SD of triplicate observations are shown in (**B**). Apoptosis was detected as ZVAD-FITC+ cells in CD3 + CD4 + population. Representative dot plots of CD4 apoptosis are shown in (**C**) and average ± SD of triplicate observations are shown in (**D**). Apoptosis was detected as ZVAD-FITC+ cells in CD3 + CD8 + population. Representative dot plots of CD8 apoptosis are shown in (**E**) and average ± SD of triplicate observations are shown (**F**). Data are expressed as mean ± SD and were compared using one-way ANOVA followed by Dunnett’s multiple comparison test. **** = *p* ≤ 0.0001.

The SupT1 and PBMCs used to assess AIP in the above experiments were of human origin. However, the KB9 and 89.6 Envs have previously been shown to cause differential pathogenesis in Rhesus Macaques. We hence evaluated whether differential AIP of SHIV 89.6 and KB9 Envs seen in Figure 7 above also translates to Rhesus PBMCs. For this, we conducted the co-culture experiments using PBMCs from healthy Rhesus Macaques as target cells. As demonstrated in Figure 8, co-culture of Rhesus PBMCs with KB9 Env expressing cells resulted in a dramatic loss of CD4 cells with a significant reduction in CD4:CD8 ratio (Figure 8A and B) compared to 89.6 Env or the pcDNA 3.1 control. Further analysis of apoptosis in different cell populations was conducted using ZVAD-FITC staining as in Figure 7 above. As seen in Figure 8C,D, there was a significant increase in CD4 apoptosis in KB9 cocultures compared to 89.6 Env or the pcDNA3.1 control. Interestingly, minimal apoptosis was detected in CD8 cells (Figure 8E,F) in all of the cocultures, further validating the specificity of Env mediated apoptosis for Rhesus CD4+ T cells as well. These results demonstrate that KB-9 Env causes specific loss of CD4 + T cells via apoptosis in Rhesus PBMC co-cultures resulting in a significant reduction in CD4:CD8 ratio.

## 4. Discussion

Several lines of evidence suggest that the Env glycoprotein remains a major determinant of HIV pathogenesis [29]. The phenotypic characteristics of HIV Env like syncytia inducing phenotype have long been associated with poor prognosis in patients [30]. Mutagenesis studies show that Envs that are deficient in cell to cell fusion fail to induce apoptosis both in vitro and in vivo in humanized mice [15,31]. Similarly, the fusogenic activity of SHIV Envs has been associated with CD4 decline in Macaque model of HIV infection [9]. Previous studies by our lab have shown that the apoptosis inducing potential or AIP of primary Envs derived from patients varies considerably [25]. More recently, we have found that this AIP of Envs correlates with CD4 decline with Envs with higher AIP associated with lower CD4 counts in patients [16]. Our in vitro assay of determining AIP could potentially serve as a surrogate for viral pathogenesis and could have prognostic value. Further validation of this phenomenon in other models like SHIV variants is warranted and forms the basis of our study.

The SHIV model of HIV infection has been extensively used for studying HIV pathogenesis. The differential pathogenesis of the SHIV variant KB9 and the parental virus SHIV 89.6 has been mapped to the Env glycoprotein [9]. More particularly, the membrane fusing activity of KB9 Env is specifically associated with pathogenesis [10]. In vitro studies with KB9 Env have also shown that the membrane fusion activity of KB9 is associated with higher syncytia formation and cytopathic affect [27]. However, it was not clear from these studies whether this cytopathic effect was related to bystander apoptosis induction. We hypothesized that our in vitro co-culture method for determination of AIP would be appropriate to test whether KB9 induces higher bystander apoptosis. Interestingly, using this model we indeed found that KB9 showed high bystander AIP both in SupT cells as well as PBMCs. In PBMC coculture experiments, KB9 showed a specific depletion of CD4 cells via apoptosis while having no effect on the CD8 T cell population. The alteration of the CD4:CD8 ratio in the PBMC coculture model was reminiscent of in vivo findings. Our findings are largely in agreement with Labonte et al. who were the first to demonstrate the cytopathic effect of KB9 Env in primary CD4 T cells [27], although their study found that the majority of cell death was occurring in CD4+ T cells and not in bystander cells. The differences in our findings are likely due to the differences in the model systems used. While we used a coculture model whereby Env is expressed on Hela cells that are cocultured with CD4+ T cells, the model by Labonte et al. involved Env expression directly in primary CD4+ T cells. Differences in the model system used for studying Env function can have a marked effect on the outcomes as shown by Cunyat et al. [32].

The Env glycoprotein of HIV is a highly complex and variable protein [33] with the two subunits gp120 and g41 having varied and distinct functions. While gp120 binds to receptor and coreceptor on target cells, the gp41 subunit mediates fusion of viral and cellular membrane [34]. As this fusion mediated by Env is a concerted effort between the functions of gp120 and gp41, variation in either of these subunits can alter Env fusogenicity. Mutations in gp120 that alter binding affinity for CD4 or coreceptor can alter Env fusion and pathogenesis [35,36,37]. Similarly, mutations in gp41 heptad repeat regions are well documented for affecting Env fusion function as well as pathogenesis [17,31,38].

Evolution of HIV Env has been extensively studied in HIV patients with late stage AIDS associated Envs being significantly different from chronic stage Envs. This evolution of Env has been correlated with phenotypic changes in Env towards increased fusogenicity, fitness and pathogenesis [39,40]. The evolution of the pathogenic SHIV KB9 from the non-pathogenic SHIV 89.6 is reminiscent of a similar evolution of the Env glycoprotein towards increased pathogenesis. Genotypic differences in SHIV KB9 and 89.6 gp120 region have been shown to be associated with differences in binding affinity for CCR5 [9]. We also find that there were differences in coreceptor usage between 89.6 and KB9 which could potentially affect pathogenicity of the viruses. Particularly, KB9 could utilize either CCR5 or CXCR4 for viral entry and apoptosis induction while 89.6 preferred CXCR4. Interestingly, Tsao et al. have demonstrated that CCR5 interaction is important for CD4 T cell depletion by a dual tropic (X4/R5) virus in humanized mice [41].

As Env fusogenicity correlates with bystander apoptosis, it is not surprising that KB9 mediated bystander apoptosis was inhibited by gp41 fusion inhibitor T20. This is consistent with other studies whereby gp41 inhibitors like T20 have been shown to inhibit Env mediated bystander apoptosis in various models [17,18,22,42]. Further support of fusion mediated by gp41 in HIV pathogenesis comes from studies using mutants that have reduced or lack gp41 mediated fusion activity and show little or no bystander apoptosis [17,31]. In support of this observation, a clinical trial of gp41 fusion inhibitor T20 (Enfuvirtide) found that resistant viruses with mutations in gp41 region were associated with increased CD4 count even after virological failure [43]. Coincidently, we found that these Enfuvirtide resistant Envs showed lower bystander apoptosis in our coculture model [31]. Our study in humanized mice using Enfuvirtide resistant gp41 mutant V38E, that lacks AIP in vitro, showed a lack of apoptosis induction in vivo and slower CD4 decline compared to WT virus in humanized mice [15]. Studies by Tsao et al. have also shown that Env variants with different coreceptor binding specificity and fusion have differential pathogenesis in humanized mice despite both viruses replicating to similar levels. [41].

The involvement of apoptosis induction in other primate models of HIV infection like SIV infection in Macaques has also been studied [44]. The non-pathogenic infection of Sooty Mangabeys with SIV_agm_ compared to pathogenic infection of Rhesus Macaques with SIV_mac251_ has in part been associated with apoptosis induction [2,45,46]. Several studies have shown that differential pathogenesis in SIV models correlates with CD4+ T cell apoptosis during early stages of the disease [3,44,47,48]. Recently, Laforge et al. [49] demonstrated the potential of a caspase inhibitor Q-VD-OPH to limit CD4 T cell apoptosis and progression to AIDS in Rhesus Macaques infected with SIV [49]. Mathematical modeling by Matrajt et al. concluded that bystander apoptosis plays a key role in CD4+T cell depletion during SHIV89.6P infection in Rhesus Macaques [50].

We find that the apoptotic signaling in bystander cells in our coculture model shows signs of classical apoptosis including mitochondrial depolarization, PS exposure, caspase activation and PARP cleavage. Moreover, studies with the pan-caspase inhibitor ZVAD-fmk demonstrate that apoptosis is at least partially inhibited via inhibition of this pathway although other mechanisms of cell death may be involved as well. Collectively, these findings support a role of bystander apoptosis induction by Env glycoprotein in CD4 loss by SHIV-KB9 Env glycoprotein. Our study also provides evidence that selective induction of apoptosis in bystander CD4+ T cells might be the mechanism behind the differential pathogenesis of SHIV 89.6 and KB9. Finally, in vitro determination of AIP of primary Envs via coculture assay might provide new insights into the determinants of HIV pathogenesis.
